# Childhood maltreatment mediates the effect of the genetic background on psychosis risk in young adults

**DOI:** 10.1038/s41398-022-01975-1

**Published:** 2022-06-01

**Authors:** Mattia Marchi, Laurent Elkrief, Anne Alkema, Willemijn van Gastel, Chris D. Schubart, Kristel R. van Eijk, Jurjen J. Luykx, Susan Branje, Stefanos Mastrotheodoros, Gian M. Galeazzi, Jim van Os, Charlotte A. Cecil, Patricia J. Conrod, Marco P. Boks

**Affiliations:** 1 Department of Biomedical, Metabolic and Neural Sciences, University of Modena and Reggio Emilia, Via Giuseppe Campi, 287 – 41125 Modena, Italy; 2 Department of Psychiatry, Brain Center University Medical Center Utrecht, University Utrecht, Utrecht, The Netherlands; 3 Department of Psychiatry, Université de Montréal, CHU Sainte-Justine Hospital, Montréal, QC Canada; 4 Center Hospitalier Universitaire Sainte-Justine Research Center, Montreal, QC Canada; 5 Department of Psychology, Utrecht University, Utrecht, The Netherlands; 6 Department of Psychiatry, Tergooi Hospital, Blaricum, The Netherlands; 7 Department of Neurology and Neurosurgery, UMC Utrecht Brain Center, Utrecht University, Utrecht, The Netherlands; 8 Department of Youth and Family, Faculty of Social and Behavioral Sciences, Utrecht University, Utrecht, The Netherlands; 9 Department of Psychology, University of Crete, Rethymno, Greece; 10 Department of Mental Health and Addiction Services, Azienda USL-IRCCS di Reggio Emilia, Reggio Emilia, Italy; 11 Department of Child and Adolescent Psychiatry, Erasmus Medical Center, Sophia Children’s Hospital, University Medical Center Rotterdam, Rotterdam, The Netherlands; 12 Department of Epidemiology, Erasmus Medical Centre, Rotterdam, The Netherlands

**Keywords:** Genetics, Psychiatric disorders

## Abstract

Childhood maltreatment (CM) and genetic vulnerability are both risk factors for psychosis, but the relations between them are not fully understood. Guided by the recent identification of genetic risk to CM, this study investigates the hypothesis that genetic risk to schizophrenia also increases the risk of CM and thus impacts psychosis risk. The relationship between schizophrenia polygenetic risk, CM, and psychotic-like experiences (PLE) was investigated in participants from the Utrecht Cannabis Cohort (*N* = 1262) and replicated in the independent IMAGEN cohort (*N* = 1740). Schizophrenia polygenic risk score (SZ-PRS) were calculated from the most recent GWAS. The relationship between CM, PRS, and PLE was first investigated using multivariate linear regression. Next, mediation of CM in the pathway linking SZ-PRS and PLE was examined by structural equation modeling, while adjusting for a set of potential mediators including cannabis use, smoking, and neuroticism. In agreement with previous studies, PLE were strongly associated with SZ-PRS (*B* = 0.190, *p* = 0.009) and CM (*B* = 0.575, *p* < 0.001). Novel was that CM was also significantly associated with SZ-PRS (*B* = 0.171, *p* = 0.001), and substantially mediated the effects of SZ-PRS on PLE (proportion mediated = 29.9%, *p* = 0.001). In the replication cohort, the analyses yielded similar results, confirming equally strong mediation by CM (proportion mediated = 34.7%, *p* = 0.009). Our results suggest that CM acts as a mediator in the causal pathway linking SZ-PRS and psychosis risk. These findings open new perspectives on the relations between genetic and environmental risks and warrant further studies into potential interventions to reduce psychosis risk in vulnerable people.

## Introduction

Schizophrenia is a complex phenotype, understood as a neurodevelopmental, polygenic, and multifactorial disorder [1, 2]. Although the prevalence of schizophrenia spectrum disorders is relatively low—approximately 0.47% for schizophrenia and 3.0% for other clinical diagnoses of psychotic disorders [3, 4]—these are responsible for tremendous personal, economic, and societal burden, with 218 disability adjusted life years (DALYs) per 100,000, making schizophrenia the fifth leading cause of DALYs in the 15–44 age group [5]. Psychotic symptoms are polymorphic symptomatic manifestations, including reality distortion, cognitive disturbance, and negative symptoms, typically presenting phases of remission and relapses. Crucially, psychotic symptoms can be considered on a continuum from non-clinical to clinical population [6]. On this continuum, some individuals report psychotic-like experiences (PLE) that do not cause sufficient impairment or distress to warrant a clinical diagnosis [3, 4]. However, PLE are thought to reflect the risk to psychosis and non-psychotic psychopathology, such as mood disorders, attention deficit and hyperactivity disorder, and suicidality [7–9]. Convergent evidence from observational studies identified several risk factors for PLE. These include (but are not limited to) cannabis use, childhood maltreatment (CM), and genetic risk [10–14]. In addition to a direct genetic basis of PLE, genetic risk may act by making individuals more sensitive to the effects of environmental exposures, such as cannabis use [15], or alternatively by driving individuals to higher exposure rates, such as the propensity to use cannabis [16]. Evidence that genetic risks are related to environmental exposures, thereby producing gene–environment correlations (rGE), are mounting [17–19]. Recent studies have identified genetic risks to CM and proposed causal relations between CM and severe mental health conditions, including schizophrenia [20, 21] and a first study found that schizophrenia risk genes can predict poorer child mental health, through increased exposure to CM [22].

Considering the importance of CM as a risk factor for psychosis and the importance of PLE as an indicator of the “high-risk” state for developing psychosis in the general population, we investigated the relationship between genetic risk to schizophrenia, CM, and PLE in two independent cohorts. To account for other relevant environmental exposures, we extended the analyses to cannabis use and tobacco smoking. To gain further insight into whether any of the identified associations are specific to maltreatment subtypes, we also modeled the subscores of our CM assessment (emotional, physical abuse and neglect, and sexual abuse subscores of the Childhood Trauma Questionnaire (CTQ)). In order to alleviate concerns about the self-reporting of CM, we also investigated the role of neuroticism that has previously been linked to the propensity to endorse trauma-related questions [23, 24].

We hypothesize that CM and cannabis use act as mediators in the pathway linking the genetic liability to schizophrenia to PLE. Such an rGE influence could have implications for the way genetic risk to psychopathology can be viewed and also – in theory - provide new perspectives on targeted interventions and prevention.

## Materials and methods

### Study design and participants—discovery sample

The Utrecht Cannabis Cohort (UCC) consists of 1262 Dutch young adults of European ancestry aged from 18 to 25 years, recruited using a project website launched in 2006 [25]. A selective sampling strategy aimed at increasing the power for GxE detection was implemented, as reflected by higher cannabis use and PLE levels in UCC [26, 27]. Data on genetics, PLE, socio-demographics, and cannabis use were fully complete. The exposure to CM was assessed through the self-reported CTQ [28]. Participants gave written informed consent, and the study was approved by the University Medical Centre Utrecht medical ethical committee.

### Study design and participants—replication sample

The IMAGEN cohort is a longitudinal imaging genetics study of over 2000 non-clinical adolescents, mostly of European descent. Detailed descriptions of this study, genotyping procedures, and data collection have previously been published [29]. The current study uses data from 1740 participants, all of the European ancestry, who contributed their genetic data and completed the PLE measure. The multicentric IMAGEN project had obtained ethical approval by the local ethics committees (at their respective sites) and written informed consent from all participants and their legal guardians.

### Genetic data and PRS

Polygenic risk scores for schizophrenia (SZ-PRS) were calculated for each of the UCC and IMAGEN individuals, who passed genetic QC. Only autosomes were included in the calculation of PRS [30]. SZ-PRS were built using data from the most recent schizophrenia GWAS based on 40,675 cases and 64,643 controls [31] as a training set. PRS were calculated using PRSice2 [32]. For each individual, SZ-PRS was calculated using 13 different *p* value thresholds (pt): 5 × 10^−8^, 5 × 10^−7^, 5 × 10^−6^, 5 × 10^−5^, 5 × 10^−4^, 5 × 10^−3^, 5 × 10^−2^, 0.5, 0.4, 0.3, 0.2, 0.1, 1. From these thresholds, one optimal threshold was selected. First, a LASSO regression was fitted to identify which SZ-PRS pt constituted the best predictor of PLE within the sample, while adjusting for age, gender, and the first three principal components. This approach has been shown to be the best way to select the right predictor from a set of variables [33]. In case of multiple PRS pt identified by the LASSO, the linear regression model’s variance explained (i.e., the *R*^2^) was used to select the SZ-PRS pt with the highest explained variance.

### Assessments

In both the discovery and replication samples, CM was assessed using the 25-item version of the CTQ [28]. The CTQ assesses self-reported CM consisting of emotional abuse, physical abuse, sexual abuse, emotional neglect, and physical neglect. The validity of the CTQ has been demonstrated in clinical and community samples [28, 34]. The CTQ continuous sum score was used as a measure of CM.

Neuroticism was assessed both in the discovery and the replication sample using the neuroticism scale of the NEO Personality Inventory [35], a validated 240-items self-reported questionnaire examining a person’s big five personality traits [36].

In the discovery sample the self-reported lifetime cannabis exposure was measured on a scale ranging from never, 1, 2, 5–9, ≥10 times. The possible concomitant use of recreational drugs and tobacco was assessed with the substance-abuse module of the Composite International Diagnostic Interview [37]. In the replication sample, participants were repeatedly assessed for cannabis use using the European School Survey of Alcohol and other Drugs (ESPAD) questionnaire at 14, 16, 18, and 21 years of age. The ESPAD is a self-report questionnaire that measures use of various drugs, including cannabis and tobacco [38]. Cannabis use data were drawn from the 18-year-old follow-ups and categorized based on the lifetime frequency of reported exposure (as never, “1–2”, “3–5”, “6–9”, “10–19”, “≥20” times). To allow for direct comparison between the two cohorts, cannabis use data were dichotomized into “case/control” status, where ≥10 lifetime uses are considered cases.

The Community Assessment of Psychic Experiences (CAPE) was used to assess psychotic experiences in both cohorts. This validated 42-items self-report questionnaire measures the prevalence of psychotic experiences on a frequency scale ranging from “never” (1), “sometimes” (2), “often” (3), to “nearly always” (4). The CAPE displays discriminative validity in assessing psychotic experiences in the general population [39]. The continuous total score of the scale was used as a measure of PLE.

### Statistical analyses

First, multivariate linear regression was used to examine the relationship between PLE, as the outcome, and CM and SZ-PRS, as main determinants, alongside age and gender as covariates.

For mediation analysis, we obtained the variance/covariance matrix (available as Supplementary Table 1), calculating the correlation among each variable. Then we performed parallel multiple mediation analysis using maximum likelihood estimation (MLE) path analysis to assess the effect of SZ-PRS on PLE directly and indirectly through the postulated mediators. The bias-corrected bootstrap 95% confidence interval (95% CI) for the indirect effect was tested to be entirely above zero based on 5000 bootstrap samples applying the adjusted bootstrap percentile method (BCa). The path diagrams were built following the structural equation modeling graphing convention (details in the Supplementary information). Statistical analyses were performed with R, using the packages “glmnet” for the LASSO [40], “lavaan” [41], and “tidySEM” [42] for the mediation analysis.

## Results

### Description of the sample(s)

The main characteristics of both the discovery and replication samples are displayed in Table 1. There was no significant difference in the male/female ratio between the two cohorts (*p* = 0.986). UCC participants were older (*p* < 0.001), more exposed to cannabis (*p* < 0.001), and reported more PLE (*p* < 0.001) than those from IMAGEN which, however, reported higher exposure to CM (*p* < 0.001) and tobacco (*p* < 0.001).

**Table 1:** Characteristics of the discovery sample and the replication sample.

Variable	Discovery sample UCC (*N* = 1262)	FMI	Replication sample IMAGEN (*N* = 1740)	FMI
Male gender (%)	606 (48%)		813 (47%)	
Age, mean (SD) in year	20.5 (2.5)		19.4 (1.5)	
CTQ score, mean (SD)	31.9 (8.4)	0.23	40.2 (6.5)	0.12
Cannabis exposure ≥10 lifetime (%)	404 (32%)	0	361 (21%)	0.063
Tobacco daily consumption for at least 30 days in the last year (%)	227 (30%)	0.003	594 (34%)	0.024
CAPE-42 score, mean (SD)	67.3 (13.9)	0	60.6 (11.4)	0

*FMI* fraction of missing information, *SD* standard deviation, *CTQ* Childhood Trauma Questionnaire, *CAPE* The Community Assessment of Psychic Experiences.

### Missing data handling

We used multiple imputation (MI) algorithm as automated in the lavaan package by the option missing = “ML” [41] to handle missing data. In the UCC, there were 341 response items missing to CTQ; in the IMAGEN cohort, there were 234 missing data about cannabis use status, and 396 missing response items to CTQ. All these missing information were imputed using case-wise MLE. MI has shown to be the most robust way to handle missing data also in case of high missing rate (even up to 50%) [43, 44]. To investigate the influence of the MI on our results, we ran the analysis also using listwise deletion and the results were fully retained (see Supplementary Table 2 and Supplementary Fig. 1).

### PRS p-threshold selection

In order to focus on PLE as the main outcome, we selected optimal PRS-SZ *p* value threshold by LASSO regression, which selected 3 out of 13 PRS-SZ pt as the best predictors of PLE in the UCC. As can be seen in Supplementary Table 3, the highest explained variance for PLE was with SZ-PRS pt 0.5 (*R*^2^ = 0.014), which was selected as the indicator of the genetic risk of schizophrenia in the subsequent analyses.

### Linear regression

From the multivariate linear regression, both CM and SZ-PRS significantly increased the level of PLE in the UCC (unstandardized regression beta [*B*] = 0.644, *p* < 0.001; *B* = 0.253, *p* = 0.003, respectively), above and beyond the influence of age and gender.

### Multiple mediation analysis in the discovery sample (UCC)

From the multiple mediation analysis conducted in the UCC, the genetic vulnerability to schizophrenia influenced the level of PLE both directly and indirectly through its effect on CM. Furthermore, cannabis use was a significant mediator too.

**Fig. 1:**
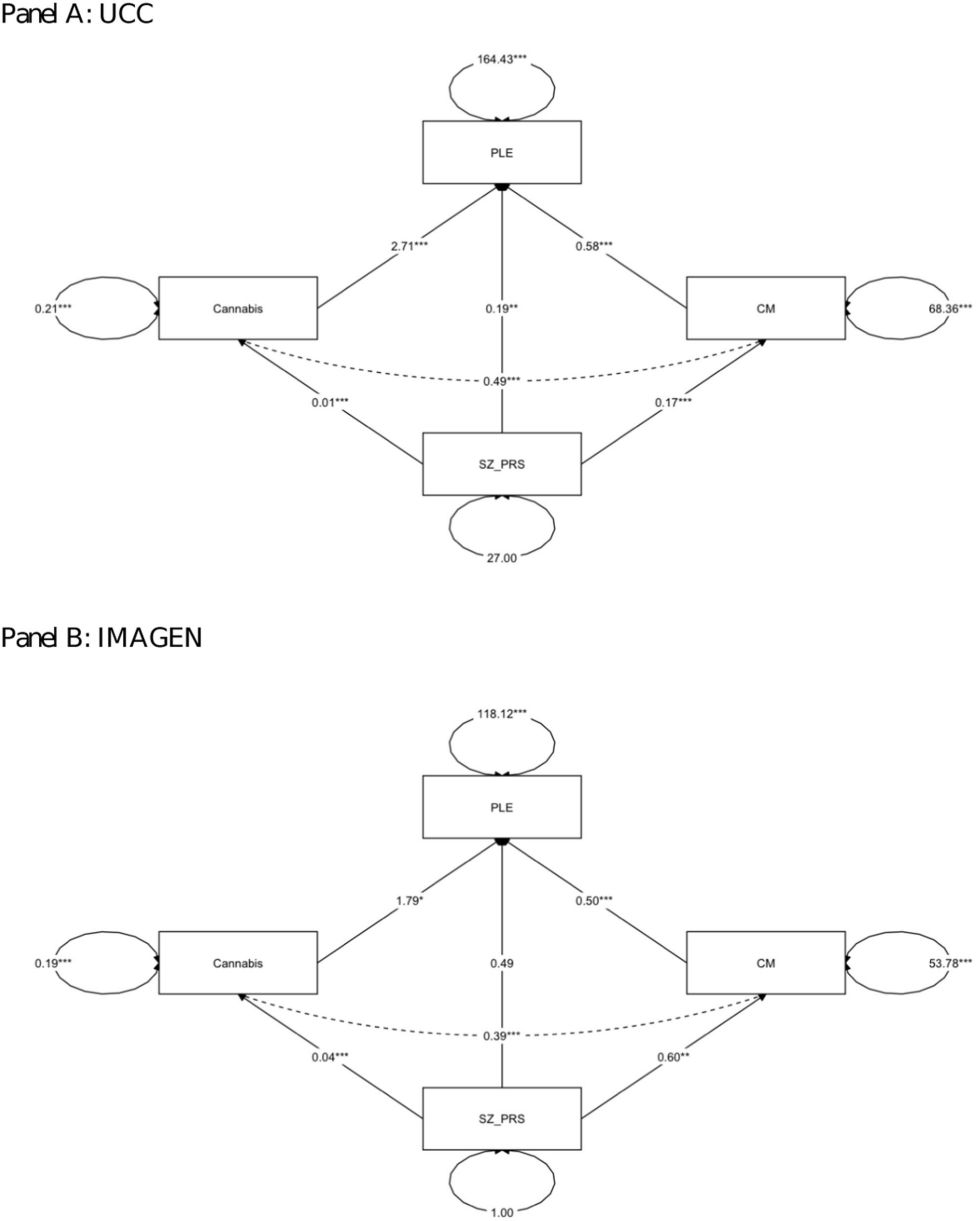
Path diagram of parallel multiple mediator model in the discovery sample and in the replication sample. Panel **A** UCC, above; Panel **B** IMAGEN cohort, below. The estimates reported are the unstandardized regression coefficients. *p < 0.05; **p < 0.01; ***p < 0.001. SZ-PRS schizophrenia polygenic risk score at *p* value threshold 0.5, CM childhood maltreatment, PLE psychotic-like experiences.

Table 2 and Fig. 1A show that subjects with higher load of SZ-PRS experienced more PLE (*B* = 0.190), and subjects who were exposed to CM and cannabis reported more PLE (*B* = 0.575, and *B* = 2.71, respectively). The bootstrap 95% CIs for the indirect effect of both CM (*B* = 0.098) and cannabis use (*B* = 0.040) were entirely above zero (0.039–0.158, and 0.013–0.067, respectively), consistent with a significant contribution of SZ-PRS to PLE through cannabis and CM.

**Table 2:** Results of parallel multiple mediator model in the discovery sample (UCC—above) and in the replication sample (IMAGEN cohort—below).

Regressions	Unstandardized *B* estimate (95% CI)	Standardized estimate	*p* value
*UCC*			
Direct effects			
Outcome model: PLE (*R*^2^ = 0.148)			
SZ-PRS	0.190 (0.048; 0.331)	0.071	0.009
CM	0.575 (0.480; 0.671)	0.344	<0.001
Cannabis use	2.71 (1.12; 4.30)	0.091	0.001
Mediator model: CM (*R*^2^ = 0.011)			
SZ-PRS	0.171 (0.072; 0.270)	0.107	0.001
Mediator model: cannabis use (*R*^2^ = 0.027)			
SZ-PRS	0.015 (0.010; 0.020)	0.164	<0.001
Covariances			
CM—cannabis use	0.489 (0.248; 0.730)	0.129	<0.001
Indirect effects (proportion mediated)			
CM (29.9%)	0.098 (0.039; 0.158)	0.037	0.001
Cannabis (12.2%)	0.040 (0.013; 0.067)	0.015	0.004
Sum	0.138 (0.073; 0.204)	0.052	<0.001
Total effect	0.328 (0.181; 0.474)	0.123	<0.001
*IMAGEN cohort*			
Direct effects			
Outcome model: PLE (*R*^2^ = 0.114)			
SZ-PRS	0.489 (−0.145; 1.12)	0.042	0.131
CM	0.496 (0.402; 0.589)	0.316	<0.001
Cannabis use	1.79 (0.302; 3.27)	0.067	0.018
Mediator model: CM (*R*^2^ = 0.007)			
SZ-PRS	0.603 (0.170; 1.04)	0.082	0.006
Mediator model: cannabis use (*R*^2^ = 0.009)			
SZ-PRS	0.041 (0.017; 0.065)	0.095	0.001
Covariances			
CM—cannabis use	0.389 (0.191; 0.588)	0.123	<0.001
Indirect effects (proportion mediated)			
CM (34.7%)	0.299 (0.076; 0.522)	0.026	0.009
Cannabis (8.6%)	0.074 (−0.001; 0.149)	0.006	0.054
Sum	0.373 (0.135; 0.611)	0.032	0.002
Total effect	0.862 (0.200; 1.52)	0.075	0.011

*95% CI* 95% bias-corrected bootstrap confidence interval, *UCC* Utrecht Cannabis Cohort, *PLE* psychotic-like experiences, *CM* childhood maltreatment, *SZ-PRS* schizophrenia polygenic risk score at *p* value threshold 0.5.

### Multiple mediation analysis in the replication sample (IMAGEN cohort)

Table 2 and Fig. 1B show the parallel multiple mediator model in the independent IMAGEN cohort. The indirect effect of SZ-PRS through CM (*B* = 0.299) was confirmed (bootstrap 95% CI: 0.076; 0.522). In contrast to the findings in the discovery set, neither the direct (*B* = 0.489; *p* = 0.131) nor the indirect effect through cannabis use (*B* = 0.074 [bootstrap 95% CI: −0.001; 0.149]) were replicated, which imply a full mediation played by CM in the replication IMAGEN cohort.

### Sensitivity and genetic threshold of mediation results

Sensitivity analysis for the causal mediation effect of CM was conducted by calculating the correlation across the residual errors of the mediator and outcome models, defined as *ρ*, which serves as the sensitivity parameter [45]. We derived the mediation effect as a function of *ρ*, and interpreted *ρ* via the *R*^2^ for understanding the influence of potential omitted variables in terms of its explanatory power (see Supplementary Figs. 2 and 3 for graphical representations). In our model, the value of *ρ* at which the causal mediation effect of CM equals zero was 0.35, meaning that to nullify our conclusions a confounder would have to explain at least 12% (*R*^2^ = 0.123) of the variance in CM thus supporting the robustness of our findings.

In order to test the robustness of our analytic procedure, and to alleviate any concerns with respect to the selection of PRS pt optimal for PLE rather than CM, we fitted the main model also for the PRS pt which best predicted CM (i.e., PRS-SZ pt 0.3). PRS-SZ pt 0.5 and pt 0.3 were highly correlated (*r* = 0.99). The results were identical, and the proportion mediated only changed at the decimal place (proportion mediated = 30.2%).

To understand at which level of genetic risk the mediation comes into play, we simulated a sample of ten times bigger size than the UCC with otherwise the same characteristics. Then, we repeated the multiple mediation analysis by stepwise removing the highest fifth percentile of genetic risk until the causal mediation effect became void (i.e., the bootstrap 95% CI included zero). This inductive approach allowed us to estimate that mediation by CM comes into play for individuals at the 60th percentile of PRS-SZ.

### Exploring other models and confounder analysis

**Fig. 2:**
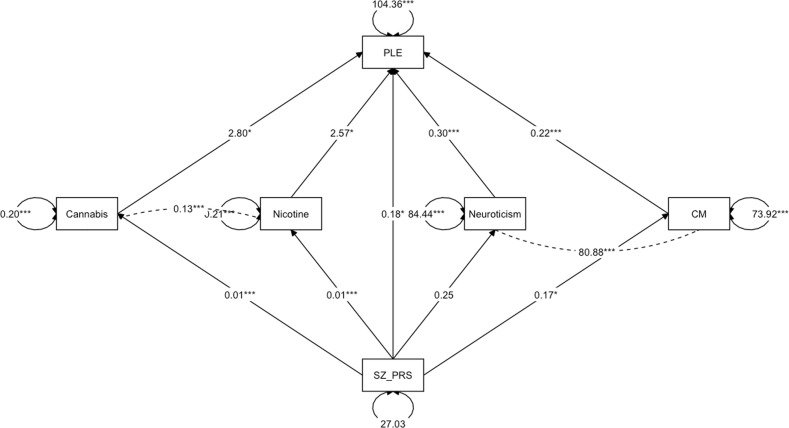
Path diagram of parallel four-mediator model in the discovery sample (UCC). The estimates reported are the unstandardized regression coefficients. *p < 0.05; **p < 0.01; ***p < 0.001. SZ-PRS schizophrenia polygenic risk score at *p* value threshold 0.5, CM childhood maltreatment, PLE psychotic-like experiences.

To investigate the impact of potential confounders, some complementary models were assessed. Specifically, we aimed to account for neurotic traits of personality—since these may affect the self-reporting in questionnaires [21]—and tobacco smoking, given the strong correlation with cannabis use and previous studies showing its impact on PLE [46, 47]. As shown in Table 3 and Fig. 2, from the four-mediator path analysis, the SZ-PRS influenced the level of PLE both directly (*B* = 0.187) and indirectly through CM and cannabis exposure (*B* = 0.038 [bootstrap 95% CI: 0.003; 0.073] and *B* = 0.035 [bootstrap 95% CI: 0.002; 0.068], respectively). Although SZ-PRS influenced nicotine exposure (*B* = 0.012) and neurotic traits influenced the self-reporting of PLE (*B* = 0.302), overall, there was no evidence for mediation by nicotine and neuroticism (*B* = 0.029 [bootstrap 95% CI: −0.002; 0.059] and *B* = 0.075 [bootstrap 95% CI: −0.036; 0.186], respectively). Also, the reported mediating role of CM was largely unchanged.

**Table 3:** Results of parallel four-mediator model in the discovery sample (UCC).

Regressions	Unstandardized *B* estimate (95% CI)	Standardized estimate	*p* value
*Direct effects*			
Outcome model: PLE (*R*^2^ = 0.424)			
SZ-PRS	0.187 (0.035; 0.339)	0.072	0.016
CM	0.223 (0.117; 0.328)	0.143	<0.001
Cannabis use	2.78 (0.538; 5.02)	0.094	0.015
Neuroticism	0.302 (0.265; 0.338)	0.541	<0.001
Nicotine	2.57 (0.168; 4.61)	0.082	0.035
Mediator model: CM (*R*^2^ = 0.011)			
SZ-PRS	0.172 (0.036; 0.307)	0.103	0.013
Mediator model: cannabis use (*R*^2^ = 0.021)			
SZ-PRS	0.013 (0.006; 0.019)	0.144	<0.001
Mediator model: neuroticism (*R*^2^ = 0.003)			
SZ-PRS	0.247 (−0.120; 0.614)	0.053	0.186
Mediator model: nicotine (*R*^2^ = 0.018)			
SZ-PRS	0.012 (0.006; 0.018)	0.136	<0.001
*Covariances*			
CM—cannabis use	0.389 (0.065; 0.713)	0.101	0.018
CM—neuroticism	80.89 (62.81; 98.97)	0.390	<0.001
CM—nicotine	0.149 (−0.183; 0.481)	0.038	0.379
Cannabis—nicotine	0.129 (0.112; 0.146)	0.628	<0.001
Cannabis—neuroticism	0.080 (−0.791; 0.951)	0.007	0.858
Neuroticism—nicotine	0.472 (−0.423; 1.37)	0.043	0.301
*Indirect effects (proportion mediated)*			
CM (10.5%)	0.038 (0.003; 0.073)	0.015	0.033
Cannabis (9.6%)	0.035 (0.002; 0.068)	0.013	0.038
Neuroticism (20.7%)	0.075 (−0.036; 0.186)	0.029	0.188
Nicotine (8.0%)	0.029 (−0.002; 0.059)	0.011	0.065
Sum	0.176 (0.045; 0.308)	0.068	0.009
*Total effect*	0.363 (0.181; 0.545)	0.140	<0.001

*95% CI* 95% bias-corrected bootstrap confidence interval, *PLE* psychotic-like experiences, *CM* childhood maltreatment, *SZ-PRS* schizophrenia polygenic risk score at *p* value threshold 0.5.

Finally, to investigate the influence of other potential confounders on the relationship between SZ-PRS and PLE, we examined the role of population stratification (using the first three genetic principal components [PC1-3]), age, and gender. We used a two-step procedure, whereby firstly we calculated the correlation between the potential confounders and the outcome (i.e., PLE) and the main predictor (i.e., SZ-PRS). Those displaying a correlation coefficient higher than 0.7, or *p* value <0.05, were subsequently included in a linear model to check whether the predictor’s beta coefficient changed more than 10%, suggesting that the variable in question is likely a confounder, that needs to be controlled. The only variable that passed the first step was PC1, which resulted significantly associated with SZ-PRS (*p* < 0.001), but affected the beta <10%, so it was not included in the models.

### Exploring CM types

We explored which type of CM (based on subscores of the CTQ) was the strongest mediator of the relationship between SZ-PRS and the level of PLE. Results are displayed in Supplementary Fig. 5 and Supplementary Table 5: emotional abuse had the largest effect size on PLE (*B* = 1.49), and also relevant association with SZ-PRS (*B* = 0.038) but did not significantly mediate the effect of SZ-PRS on PLE (*B* = 0.057 [bootstrap 95% CI: −0.002; 0.117]). Only emotional neglect was a significant mediator in the relationship between SZ-PRS and PLE (*B* = 0.037 [bootstrap 95% CI: 0.007; 0.068]).

As can be seen in Supplementary Tables 4 and 6 and Supplementary Figs. 4 and 6, the results from the secondary analyses performed in the replication sample were similar, with the exception of tobacco, which was negatively associated with PLE. However, accounting for the mediation played by tobacco resulted in an increased significance of the indirect effect of SZ-PRS on PLE through cannabis use (*B* = 0.097 [bootstrap 95% CI: 0.017; 0.177]).

## Discussion

This study demonstrated that schizophrenia genetic risk (SZ-PRS) is associated with a greater exposure to CM and PLE in a cohort of partly selectively sampled cases of young adults (*N* = 1262) and replicated the associations of CM with SZ-PRS and PLE in a more typical population-based, independent sample (*N* = 1740). Although the direct effect of SZ-PRS on PLE did not replicate in the IMAGEN cohort (possibly due to the selection of the PRS threshold optimal for PLE in UCC), CM did mediate the effect of schizophrenia genetic risk on PLE in both cohorts, supporting the relevance of the mediation by CM even beyond differences in sampling.

Our data suggest that around 15% of PLE can be explained by our set of predictors (SZ-PRS, CM, and cannabis use), and up to one-third of the effect of schizophrenia genes on PLE is exerted through increased exposure to CM. This indicates synergy between genes and environment in shaping mental health phenotypes, such as those linked to a high risk of psychosis [14, 48–51].

The reported relationship of CM with PLE echoes previous research showing that CM is associated with multidimensional psychopathology in the general population [52–56]. Our data confirm that CM is one of the strongest components of the exposome (the dense network of environmental exposures) that contribute to psychosis proneness [57, 58]. From that perspective, the observation that CM is also associated with SZ-PRS in two independent, population-based, cohorts is very relevant. Previous research already reported a link between SZ-PRS and CM, with an indirect influence on emotional, attention, and thought problems in children [22]. The current study extends the relationship between SZ-PRS and CM to early adulthood and finds evidence of a role as mediator of genetic risk for CM. Moreover, the proportion of the effect of SZ-PRS on PLE mediated by CM in our study is much higher compared to that for emotional and cognitive problems reported by Bolhuis et al. [22]. These findings are consistent with a pleiotropic influence of schizophrenia genes. From a clinical perspective, PLE may be viewed as an important indicator of mental health vulnerability that results from the presence of schizophrenia genes, which contributions are both direct and through several pathways/mechanisms, among which we collected the strongest evidence for contributions by CM and cannabis.

The reported mediations by CM and cannabis use are robust to the adjustment for other candidate mediators, including neuroticism and tobacco smoking. Adjustment for personality traits is relevant as they have been demonstrated to influence self-reported measures. Particularly, neuroticism has been suggested to increase the likelihood of individuals to recall CM [23, 24]. However, adjusting the model for the potential influence of neuroticism did not change the role played by CM. If more frequent reporting of PLE was entirely due to high neuroticism, adding neuroticism to the model would have attenuated or even nullified our findings. Our data suggest that the mediations do not act through biased reporting, but instead are likely to reflect truly increased levels of PLE.

In agreement with previous studies, we confirm that tobacco smoking is related to PLE and cannabis use [46, 47, 59]. However, the adjustment for nicotine exposure did not alter the mediation by CM. Unexpectedly, the effect of tobacco smoking on PLE had opposite directions in the two cohorts: in the discovery sample, tobacco consumption increased PLE, similarly to cannabis; in the replication sample tobacco consumption resulted in less PLE, yet the adjustment increased the indirect effect of SZ-PRS on PLE through cannabis use. The reason for the discrepancies in the role of tobacco between the cohorts is unclear. It may be attributable to the different patterns of use between countries [59, 60] but, in the absence of further data, any speculation is premature.

By comparing CM subtypes, we found that emotional neglect (in the UCC) and emotional abuse (in the IMAGEN) are the strongest mediators of the relationship between the genetic risk of schizophrenia and PLE. That evidence complements previous findings from prominent GxE research that placed these maltreatment types among the most relevant exposures for the development of schizophrenia in people with a high load of SZ-PRS [11]. Emotional abuse was also identified as a particularly strong risk factor for PLE in studies of high-risk populations [61, 62]. However, the most plausible explanation for the strong role of emotional abuse (and to lesser extent neglect) may be that these types of CM are the most frequently endorsed and its statistical distribution translates into a higher power.

### Limitations

This study should be interpreted in light of its limitations. First, all participants originate from Western countries, which may reduce the generalizability of the findings. Second, the cross-sectional design limited the capability to address causality, and the implementation of a rigorous multiple mediation path analysis approach might have only partially bridged this constrain. Also, residual confounding cannot be ruled out. Future research should adopt prospective designs, particularly assessing the transition rates from a non-clinical high-risk state to clinical phenotypes. Third, the selection of a retrospective self-reported measure of trauma is always of concern even though the use of a frequently used validated tool, with verification questions, and the analysis of neuroticism (that may influence reporting) may have partially alleviated this problem. Fourth, the measurement scales for cannabis use are different across the two cohorts, and due to its peculiar—non-Gaussian—distribution in the population (i.e., many people never used it or used it infrequent, whereas frequent users pose a separate group leading to a bivariate distribution due to a lack of representation of the “middle part” of the distribution), to allow for direct comparison between the two cohorts, cannabis use data were dichotomized at the only possible splitting (i.e., ≥10 uses). The need for dichotomization may have affected the ability to replicate the role of cannabis in the presented models. Another limitation consists in the absence of a pre-registration protocol, that would have made the selection of tests, variables, and cut-offs more transparent because of a priori decisions. Finally, in line with most studies implementing PRS, our conclusions are based on small effect sizes. Even though our results are based on the latest release of the Psychiatric Genomics Consortium [63], SZ-PRS explain only a limited portion of the schizophrenia phenotype (i.e., around 7%), therefore in this study, the contribution of SZ-PRS to PLE and CM are rather modest (1.4% and 1.1%, respectively).

### Implication for research and clinical practice

To the best of our knowledge, this is the first study to report that exposure to CM is influenced by the genetic risk of schizophrenia with a final impact on PLE. Put simply, the mediation played by CM in the relationship between SZ-PRS and PLE indicates that rGE contribute to psychosis risk. Three potential rGE mechanisms can explain the relation between SZ-PRS and CM [20]; 1-passive rGE, 2-evocative rGE, and 3-active rGE. (1) Passive rGE refers to the association between a person’s genotype and the behavior of genetically related individuals. Parental mental health and parental history of CM are confirmed risk factors for maltreating offspring [64, 65]. A person’s genetic risk is reflective of parents’ genetic risk, and a higher genetic load for schizophrenia in parents might have influenced their children’s environment to increase the likelihood of CM. (2) Evocative rGE occurs when a person’s genetically driven behavior elicits responses from others. For instance: previous research highlighted sub-clinical psychiatric manifestation of emotional and behavioral problems in children with higher SZ-PRS [66–69] and that behavior may have led to more exposure by harder and more frequent punishments by caretakers. (3) Active rGE occurs when people’s life experiences are directly influenced by their own genetically determined behaviors, such as thrill-seeking or risk-taking behaviors.

Regardless the rGE mechanism involved in this study, CM account for up to 30% of the effect of SZ-PRS on PLE. Simulations in this study pointed out that in participants in the top 40% of SZ-PRS, mediation through CM becomes a significant mechanism. The question is whether such a threshold may provide guidance for tailoring preventative strategies. For instance, resources may be allocated for counseling of children and adolescents targeting those with the highest genetic risk and coaching their caretakers on positive parenting skills [70]. This might offer an opportunity for attenuating the risk of mental health disorder. Indeed, previous research confirmed that fostering a safe and stable relationship between intimate partners and between mothers and children is effective in reducing the intergenerational transmission of abuse in families [71]. Addressing parental mental health and parental history of childhood abuse in case of passive rGE would help to provide individualized support and to plan early interventions, such as early education about lifestyle and parenting skills [72]. However, considering that the contribution of SZ-PRS is low and other more intuitive factors (such as help-seeking behavior and educational problems) are likely to be stronger risk factors, preventive approaches based on SZ-PRS are not supported by our data.

Overall, our findings contribute to a better understanding of how rGE might explain behavior, ultimately leading to a higher risk state for mental illness in general and psychoses in particular. As such, it adds a new perspective to an already complicated relationship between genetic vulnerability and mental health.

## Supplementary information

Supplementary Materials

## Data Availability

The variance/covariance matrix is available in the Supplementary information.

## Code availability

The codes for reproducing the main model are available at https://github.com/MattiaMarchi/SEM_Sz-PRS-CM_PLE.git.
