# Supplementary material for: Childhood maltreatment mediates the effect of the genetic background on psychosis risk in young adults
Source: Transl Psychiatry. 2022 Jun 1;12:219. doi: 10.1038/s41398-022-01975-1 (PMC9160238; doi:10.1038/s41398-022-01975-1)
Supplement: Supplementary file 1 — Supplementary Materials [file 41398_2022_1975_MOESM1_ESM.docx]

**Supplementary Materials**

SEM graphing conventions

Table 1: variance-covariance matrix of the variables of the main model.

Table 2: Results of complete case analysis of parallel multiple mediator model in the discovery sample (UCC - above) and in the replication sample (IMAGEN cohort - below)

Figure 1: Path diagram of complete case analysis of parallel multiple mediator model in the discovery sample (Panel A. UCC above) and in the replication sample (Panel B. IMAGEN cohort, below).

Table 3: variance of PLE phenotype explained by the SZ-PRS selected from the LASSO.

Figure 2: Causal mediation effect of CM as function of the sensitivity parameter (ρ).

Figure 3: Explanatory power of a confounder on the causal mediation effect of CM.

Table 4: Results of parallel four-mediator model in the replication sample (IMAGEN cohort).

Figure 4: Path diagram of parallel four-mediator model in the replication sample (IMAGEN cohort).

Table 5: Results of parallel multiple mediation analysis according to the trauma subtype in the discovery sample (UCC).

Figure 5: Path diagram of parallel multiple mediation analysis according to trauma subtype in the discovery sample (UCC).

Table 6: Results of parallel multiple mediation analysis according to the trauma subtype in the replication sample (IMAGEN cohort).

Figure 6: Path diagram of parallel multiple mediation analysis according to the trauma subtype in the replication sample (IMAGEN cohort).

**SEM graphing conventions**

*From Van Lissa, C. J., (2019). tidySEM: Tidy structural equation modeling. R package version 0.2.1.* [*https://github.com/cjvanlissa/tidySEM/*](https://github.com/cjvanlissa/tidySEM/)

Structural equation modeling (SEM) uses some conventions to graph models visually.

Nodes

Nodes are the “shapes” in a graph. In SEM graphs, we use nodes to indicate variables.

*Observed variables*

An observed variable is a measured quantity; you have “real data” about this variable. Observed variables are also called measured variables or indicators. Visually, we indicate observed variables as rectangles.

*Latent variables*

A latent variable is something that exists (according to your theory), but that you did not measure directly. There is no column in the data for this variable. Latent variables are also called “unobserved” variables. Visually, we indicate them as ovals or circles.

Edges

Edges refer to the connections between nodes. In SEM graphs, we use edges to specify relationships between variables.

*Regression effects*

A regression effect indicates that one variable influences another variable. Regression effects are also called “paths”, “directional effects”, and “factor loadings”. In SEM graphs, we represent regression effects as single-headed arrows, with the accompanying unstandardized regression coefficient.

*Covariances*

Covariances are also referred to as “correlations” (when a graph depicts standardized coefficients), “undirected associations”, or “unexplained associations”. Visually, we indicate covariances as curved (dashed) lines without arrowheads.

*(Residual) variances*

Variables often have a variance, and in SEM we can represent this variance as a double-headed arrow that points from a variable to itself. However, this is a simplification. When a variable is endogenous (on the receiving end of one or more causal effects), it gains an additional latent variable predictor known as an “error term”; a latent variable with a loading of 1 and a mean of 0. The variance of this error term represents the “error variance”, also known as “residual variance” or “unexplained variance”. In tidySEM, all error terms are omitted, and all variances (including error variances) are displayed as double-headed arrows.

**Table 1: variance-covariance matrix of the variables of the main model**

|  | **CM** | **PLE** | **SZ-PRS** | **Cannabis** |
| --- | --- | --- | --- | --- |
| **CM** | 70.1399943 | 46.3551222 | 4.8813426 | 0.5704782 |
| **PLE** | 46.355122 | 212.538809 | 10.044700 | 1.000641 |
| **SZ-PRS** | 4.8813426 | 10.0447004 | 26.1217353 | 0.4124109 |
| **Cannabis** | 0.5704782 | 1.0006415 | 0.4124109 | 0.2217328 |

Abbreviations: CM: childhood maltreatment; PLE: psychotic-like experiences; SZ-PRS: schizophrenia polygenic risk score at p-value threshold 0.5.

**Table 2: Results of complete case analysis of parallel multiple mediator model in the discovery sample (UCC - above) and in the replication sample (IMAGEN cohort - below)**

| **Regressions** | **Unstandardized estimate (95% CI)** | **Standardized coefficient** | **p-value** |
| --- | --- | --- | --- |
| *UCC* | | | |
| **Direct effects** |  |  |  |
| *Outcome model: PLE (R^2^=0.159)* |  |  |  |
| SZ-PRS | 0.229 (0.056; 0.401) | 0.080 | 0.009 |
| CM | 0.625 (0.520; 0.730) | 0.359 | <0.001 |
| Cannabis use | 2.48 (0.600; 4.36) | 0.080 | 0.010 |
|  |  |  |  |
| *Mediator model: CM (R^2^=0.013)* |  |  |  |
| SZ-PRS | 0.187 (0.082; 0.292) | 0.114 | 0.001 |
| *Mediator model: Cannabis use (R^2^=0.029)* |  |  |  |
| SZ-PRS | 0.016 (0.010; 0.022) | 0.171 | <0.001 |
|  |  |  |  |
| **Covariances** |  |  |  |
| CM – Cannabis use | 0.493 (0.241; 0.744) | 0.128 | <0.001 |
|  |  |  |  |
| **Indirect effects (proportion mediated)** |  |  |  |
| CM (30.4%) | 0.117 (0.048; 0.185) | 0.041 | 0.001 |
| Cannabis (10.1%) | 0.039 (0.006; 0.072) | 0.014 | 0.020 |
| Sum | 0.156 (0.079; 0.233) | 0.055 | <0.001 |
| **Total effect** | 0.385 (0.202; 0.567) | 0.135 | <0.001 |
| *IMAGEN cohort* | | | |
| **Direct effects** |  |  |  |
| *Outcome model: PLE (R^2^=0.108)* |  |  |  |
| SZ-PRS | 0.360 (-0.305; 1.026) | 0.032 | 0.289 |
| CM | 0.486 (0.393; 0.579) | 0.307 | <0.001 |
| Cannabis use | 1.95 (0.374; 3.520) | 0.073 | 0.015 |
| *Mediator model: CM (R^2^=0.006)* |  |  |  |
| SZ-PRS | 0.573 (0.132; 1.013) | 0.080 | 0.011 |
| *Mediator model: Cannabis use (R^2^=0.010)* |  |  |  |
| SZ-PRS | 0.042 (0.016; 0.068) | 0.098 | 0.002 |
|  |  |  |  |
| **Covariances** |  |  |  |
| CM – Cannabis use | 0.351 (0.163; 0.540) | 0.116 | <0.001 |
|  |  |  |  |
| **Indirect effects (proportion mediated)** |  |  |  |
| CM (38.6%) | 0.278 (0.058; 0.499) | 0.024 | 0.013 |
| Cannabis (11.3%) | 0.081 (-0.002; 0.164) | 0.007 | 0.055 |
| Sum | 0.360 (0.120; 0.599) | 0.032 | 0.003 |
| **Total effect** | 0.720 (0.022; 1.418) | 0.063 | 0.043 |

Abbreviations: 95% CI: 95% bias-corrected bootstrap confidence interval; UCC: Utrecht cannabis cohort; PLE: psychotic-like experiences; CM: childhood maltreatment; SZ-PRS: schizophrenia polygenic risk score at p-value threshold 0.5.

**Figure 1: Path diagram of complete case analysis of parallel multiple mediator model in the discovery sample (Panel A. UCC above) and in the replication sample (Panel B. IMAGEN cohort, below).**

Legend: the estimates reported are the unstandardized regression coefficients. *p<0.05; **p<0.01; ***p<0.001.

Abbreviations: SZ-PRS: schizophrenia polygenic risk score at p-value threshold 0.5; CM: childhood maltreatment; PLE: psychotic-like experiences.

**Table 3: variance of PLE phenotype explained by the SZ-PRS selected from the LASSO**

| **SZ-PRS pt** | **Unstandardized B estimate (95% CI)** | **p-value** | **R^2^** |
| --- | --- | --- | --- |
| 0.2 | 0.348 (0.190; 0.506) | <0.001 | 0.0139 |
| 0.5 | 0.328 (0.181; 0.475) | <0.001 | 0.0143 |
| 1 | 0.326 (0.180; 0.471) | <0.001 | 0.0142 |

Abbreviations: SZ-PRS pt: schizophrenia polygenic risk score p-value threshold; 95% CI: 95% confidence interval.


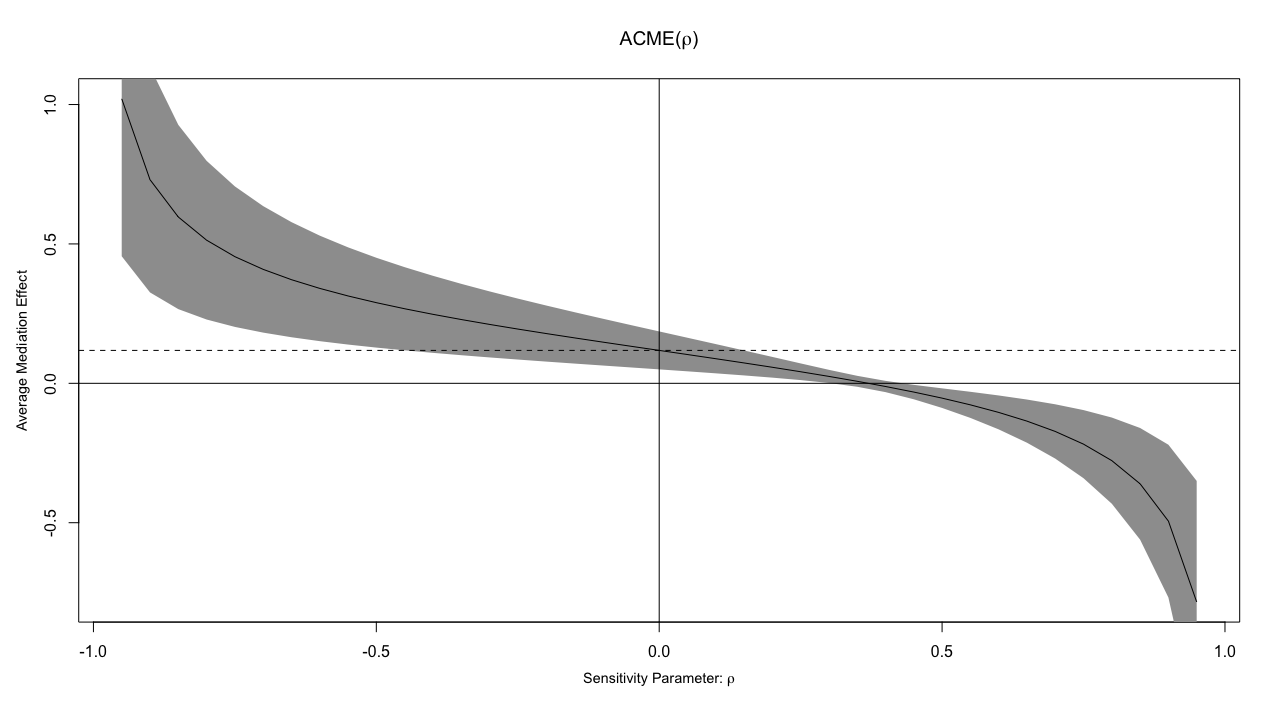


**Figure 2: Causal mediation effect of CM as function of the sensitivity parameter (ρ)**

ACME: average causal mediation effect of CM.


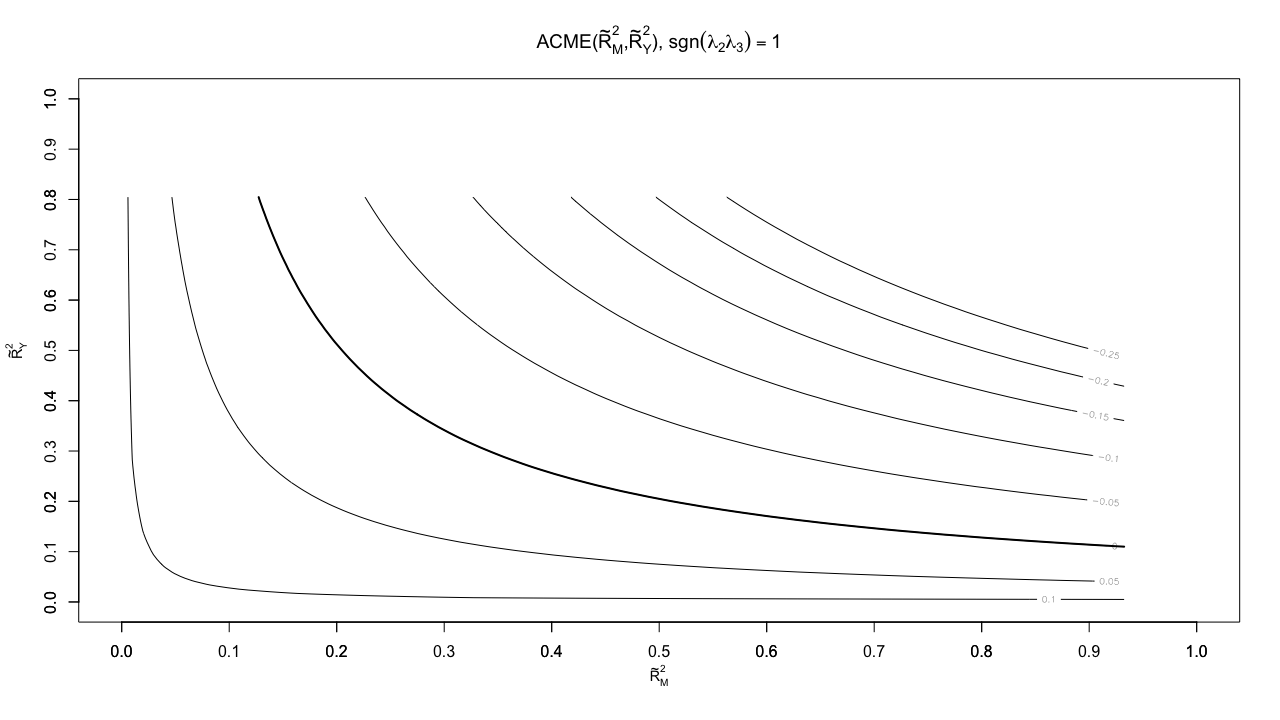


**Figure 3: Explanatory power of a confounder on the causal mediation effect of CM**

ACME: average causal mediation effect of CM

**Table 4: Results of parallel four-mediator model in the replication sample (IMAGEN cohort)**

| **Regressions** | **Unstandardized B estimate (95% CI)** | **Standardized estimate** | **p-value** |
| --- | --- | --- | --- |
| **Direct effects** |  |  |  |
| *Outcome model: PLE (R^2^=0.371)* |  |  |  |
| SZ-PRS | 0.324 (-0.207; 0.855) | 0.028 | 0.231 |
| CM | 0.270 (0.188; 0.352) | 0.172 | <0.001 |
| Cannabis use | 2.36 (0.989; 3.74) | 0.089 | 0.001 |
| Neuroticism | 0.740 (0.673; 0.806) | 0.531 | <0.001 |
| Nicotine | -1.32 (-2.53; -0.11) | -0.056 | 0.032 |
|  |  |  |  |
| *Mediator model: CM (R^2^=0.007)* |  |  |  |
| SZ-PRS | 0.618 (0.185; 1.05) | 0.084 | 0.005 |
|  |  |  |  |
| *Mediator model: Cannabis use (R^2^=0.009)* |  |  |  |
| SZ-PRS | 0.041 (0.017; 0.065) | 0.094 | 0.001 |
|  |  |  |  |
| *Mediator model: Neuroticism (R^2^=0.002)* |  |  |  |
| SZ-PRS | 0.343 (-0.117; 0.803) | 0.042 | 0.144 |
|  |  |  |  |
| *Mediator model: Nicotine (R^2^=0.001)* |  |  |  |
| SZ-PRS | 0.008 (-0.019; 0.035) | 0.016 | 0.568 |
|  |  |  |  |
| **Covariances** |  |  |  |
| CM – Neuroticism | 16.0 (12.2; 19.7) | 0.264 | <0.001 |
| Cannabis – Nicotine | 0.091 (0.078; 0.104) | 0.431 | <0.001 |
|  |  |  |  |
| **Indirect effects (proportion mediated)** |  |  |  |
| CM (20.1%) | 0.167 (0.039; 0.294) | 0.014 | 0.010 |
| Cannabis (11.6%) | 0.097 (0.017; 0.177) | 0.008 | 0.018 |
| Neuroticism (30.6%) | 0.254 (-0.087; 0.595) | 0.022 | 0.145 |
| Nicotine (-1.3%) | -0.011 (-0.048; 0.027) | -0.001 | 0.581 |
| Sum | 0.507 (0.111; 0.903) | 0.044 | 0.012 |
|  |  |  |  |
| **Total effect** | 0.831 (0.178; 1.48) | 0.072 | 0.013 |

Abbreviations: 95% CI: 95% bias-corrected bootstrap confidence interval; PLE: psychotic-like experiences; CM: childhood maltreatment; SZ-PRS: schizophrenia polygenic risk score at p-value threshold 0.5.


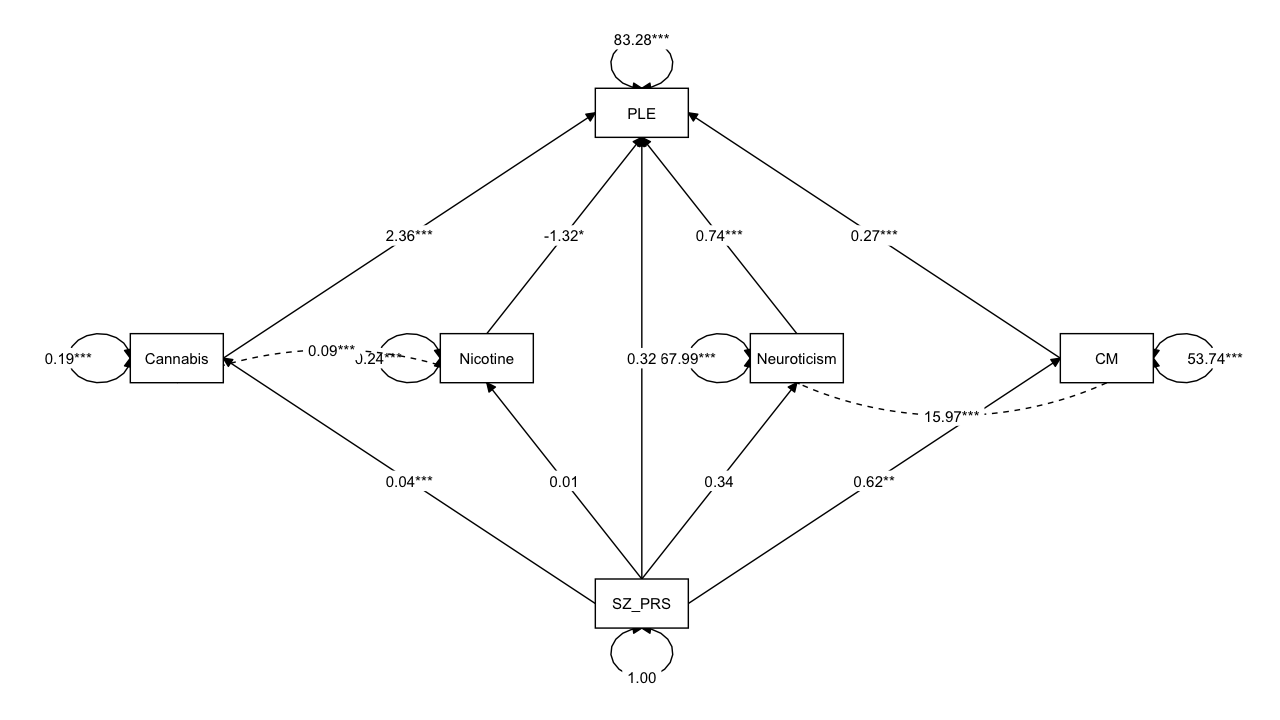


**Figure 4: Path diagram of parallel four-mediator model in the replication sample (IMAGEN cohort).**

Legend: The estimates reported are the unstandardized regression coefficients. *p<0.05; **p<0.01; ***p<0.001.

Abbreviations: SZ-PRS: schizophrenia polygenic risk score at p-value threshold 0.5; CM: childhood maltreatment; PLE: psychotic-like experiences.

**Table 5: Results of parallel multiple mediation analysis according the trauma subtype in the discovery sample (UCC)**

| **Regressions** | **Unstandardized B estimate (95% CI)** | **Standardized estimate** | **p-value** |
| --- | --- | --- | --- |
| **Direct effects** |  |  |  |
| *Outcome model: PLE (R^2^=0.168)* |  |  |  |
| SZ-PRS | 0.242 (0.102; 0.382) | 0.091 | 0.001 |
| Sexual Abuse | 0.287 (-0.137; 0.711) | 0.044 | 0.184 |
| Physical Abuse | -0.854 (-1.45; -0.257) | -0.099 | 0.005 |
| Emotional Abuse | 1.49 (1.19; 1.79) | 0.354 | <0.001 |
| Physical Neglect | 0.278 (-0.178; 0.735) | 0.036 | 0.232 |
| Emotional Neglect | 0.481 (0.221; 0.742) | 0.126 | <0.001 |
|  |  |  |  |
| *Mediator model: Sexual abuse (R^2^=0.002)* |  |  |  |
| SZ-PRS | -0.018 (-0.044; 0.008) | -0.044 | 0.176 |
|  |  |  |  |
| *Mediator model: Physical abuse (R^2^=0.007)* |  |  |  |
| SZ-PRS | 0.025 (0.006; 0.045) | 0.082 | 0.010 |
|  |  |  |  |
| *Mediator model: Emotional abuse (R^2^=0.004)* |  |  |  |
| SZ-PRS | 0.038 (-0.001; 0.078) | 0.061 | 0.006 |
|  |  |  |  |
| *Mediator model: Physical Neglect (R^2^=0.003)* |  |  |  |
| SZ-PRS | 0.019 (-0.003; 0.041) | 0.055 | 0.090 |
|  |  |  |  |
| *Mediator model: Emotional neglect (R^2^=0.012)* |  |  |  |
| SZ-PRS | 0.077 (0.029; 0.126) | 0.111 | 0.002 |
|  |  |  |  |
| **Indirect effects (proportion mediated)** |  |  |  |
| Sexual abuse (1.6%) | -0.005 (-0.016; 0.006) | -0.002 | 0.346 |
| Physical abuse (7.0%) | -0.022 (-0.044; 0.001) | -0.008 | 0.060 |
| Emotional abuse (18.1%) | 0.057 (-0.002; 0.117) | 0.021 | 0.060 |
| Physical neglect (1.6%) | 0.005 (-0.005; 0.016) | 0.002 | 0.326 |
| Emotional neglect (11.7%) | 0.037 (0.007; 0.068) | 0.014 | 0.017 |
| Sum | 0.073 (0.003; 0.134) | 0.027 | 0.041 |
|  |  |  |  |
| **Total effect** | 0.315 (0.169; 0.461) | 0.188 | <0.001 |

Abbreviations: 95% CI: 95% bias-corrected bootstrap confidence interval; PLE: psychotic-like experiences; SZ-PRS: schizophrenia polygenic risk score at p-value threshold 0.5.

**
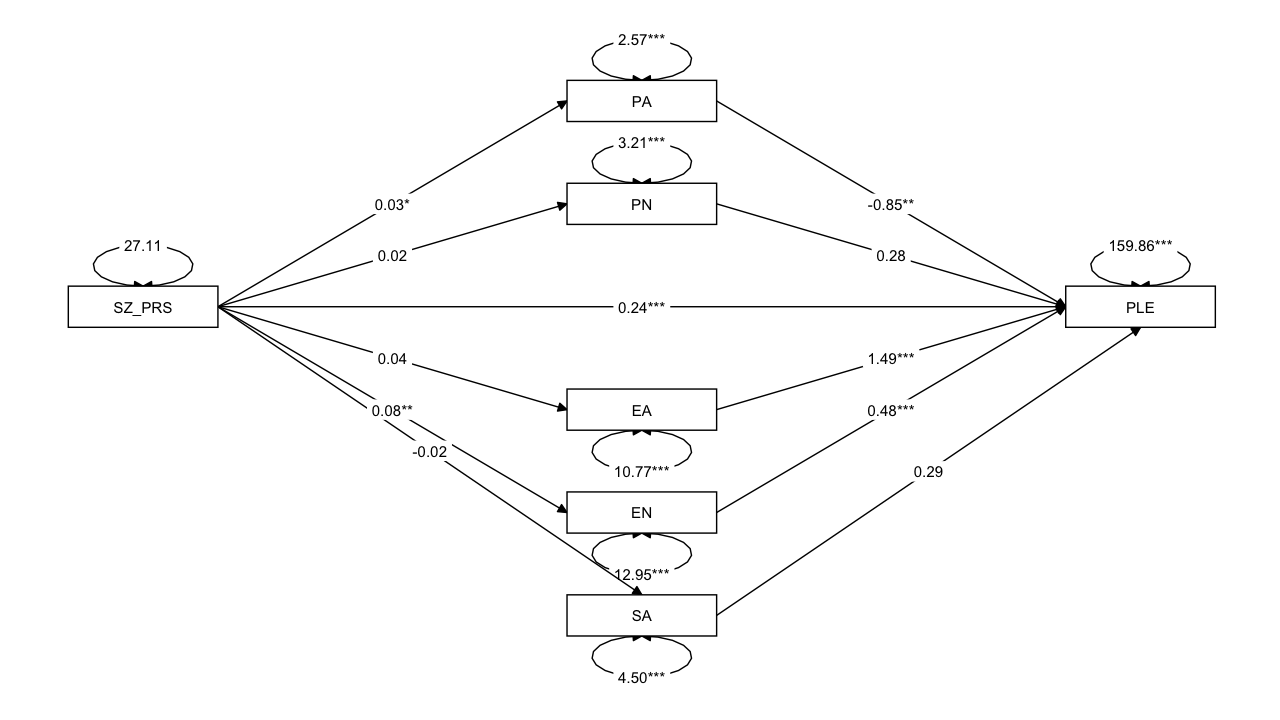
Figure 5: Path diagram of parallel multiple mediation analysis according to trauma subtype in the discovery sample (UCC).**

Legend: The estimates reported are the unstandardized regression coefficients. *p<0.05; **p<0.01; ***p<0.001.

Abbreviations: SZ-PRS: schizophrenia polygenic risk score at p-value threshold 0.5; PLE: psychotic-like experiences; PS: physical abuse; PN: physical neglect; EA: emotional abuse; EN: emotional neglect; SA: sexual abuse.

**Table 6: Results of parallel multiple mediation analysis according the trauma subtype in the replication sample (IMAGEN cohort)**

| **Regressions** | **Unstandardized B estimate (95% CI)** | **Standardized estimate** | **p-value** |
| --- | --- | --- | --- |
| **Direct effects** |  |  |  |
| *Outcome model: PLE (R^2^=0.123)* |  |  |  |
| SZ-PRS | 0.499 (-0.128; 1.13) | 0.043 | 0.119 |
| Sexual abuse | 0.046 (-0.396; 0.488) | 0.006 | 0.840 |
| Physical abuse | -0.684 (-1.45; 0.085) | -0.068 | 0.081 |
| Emotional abuse | 1.30 (0.947; 1.66) | 0.307 | <0.001 |
| Physical neglect | -0.183 (-0.669; 0.302) | -0.026 | 0.459 |
| Emotional neglect | 0.496 (0.200; 0.792) | 0.133 | 0.001 |
|  |  |  |  |
| *Mediator model: Sexual abuse (R^2^<0.001)* |  |  |  |
| SZ-PRS | 0.024 (-0.071; 0.119) | 0.015 | 0.619 |
|  |  |  |  |
| *Mediator model: Physical abuse (R^2^<0.001)* |  |  |  |
| SZ-PRS | 0.013 (-0.054; 0.081) | 0.012 | 0.699 |
|  |  |  |  |
| *Mediator model: Emotional abuse (R^2^=0.005)* |  |  |  |
| SZ-PRS | 0.184 (0.024; 0.343) | 0.068 | 0.024 |
|  |  |  |  |
| *Mediator model: Physical neglect (R^2^=0.002)* |  |  |  |
| SZ-PRS | 0.074 (-0.023; 0.171) | 0.045 | 0.136 |
|  |  |  |  |
| *Mediator model: Emotional neglect (R^2^=0.010)* |  |  |  |
| SZ-PRS | 0.305 (0.123; 0.487) | 0.099 | 0.001 |
|  |  |  |  |
| **Indirect effects (proportion mediated)** |  |  |  |
| Sexual abuse (0.1%) | 0.001 (-0.010; 0.013) | <0.001 | 0.852 |
| Physical abuse (-1.0%) | -0.009 (-0.057; 0.038) | -0.001 | 0.705 |
| Emotional abuse (27.5%) | 0.239 (0.020; 0.458) | 0.021 | 0.032 |
| Physical neglect (-1.6%) | -0.014 (-0.053; 0.026) | -0.001 | 0.505 |
| Emotional neglect (17.4%) | 0.151 (0.023; 0.280) | 0.013 | 0.021 |
| Sum | 0.369 (0.126; 0.612) | 0.032 | 0.003 |
|  |  |  |  |
| **Total effect** | 0.868 (0.211; 1.53) | 0.076 | 0.010 |

Abbreviations: 95% CI: 95% bias-corrected bootstrap confidence interval; PLE: psychotic-like experiences; SZ-PRS: schizophrenia polygenic risk score at p-value threshold 0.5.


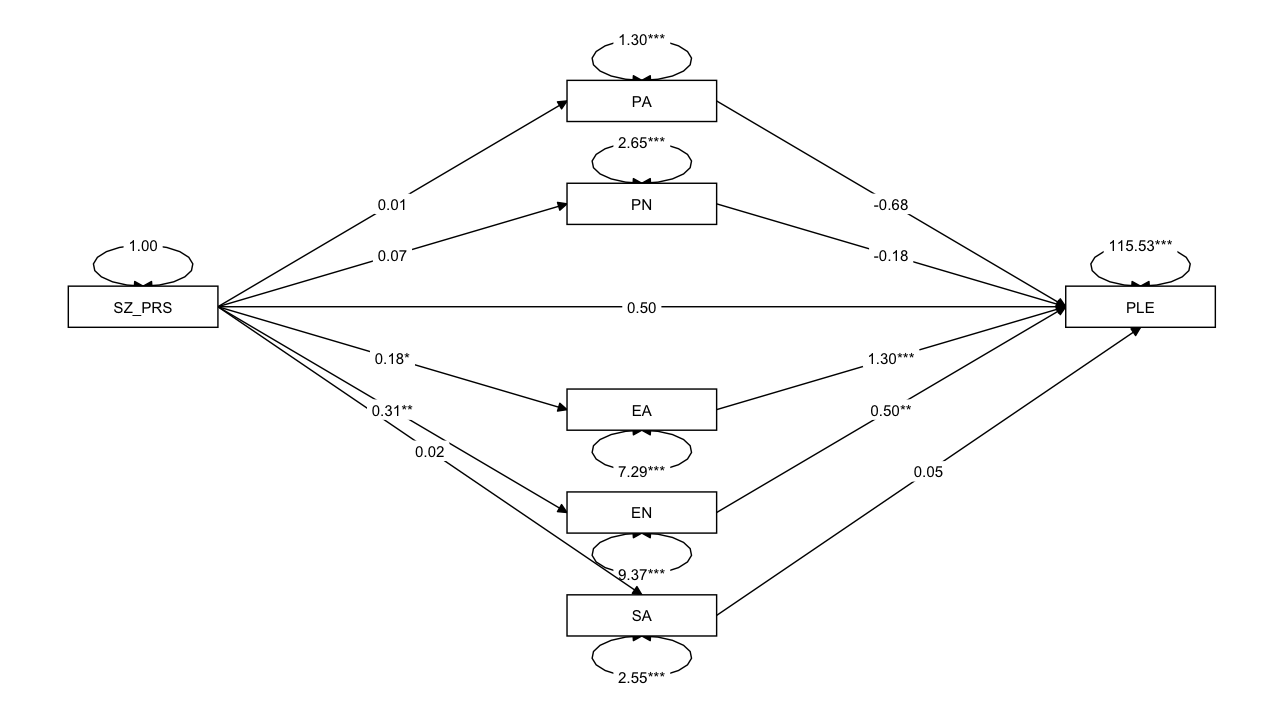


**Figure 6: Path diagram of parallel multiple mediation analysis according the trauma subtype in the replication sample (IMAGEN cohort)**

Legend: The estimates reported are the unstandardized regression coefficients. *p<0.05; **p<0.01; ***p<0.001.

Abbreviations: SZ-PRS: schizophrenia polygenic risk score at p-value threshold 0.5; PLE: psychotic-like experiences; PS: physical abuse; PN: physical neglect; EA: emotional abuse; EN: emotional neglect; SA: sexual abuse.
